# Visualisation and characterisation of mononuclear phagocytes in the chicken respiratory tract using *CSF1R*-transgenic chickens

**DOI:** 10.1186/s13567-018-0598-7

**Published:** 2018-10-10

**Authors:** Kate Sutton, Taiana Costa, Andreas Alber, Karen Bryson, Dominika Borowska, Adam Balic, Pete Kaiser, Mark Stevens, Lonneke Vervelde

**Affiliations:** Division of Infection and Immunity, The Roslin Institute and Royal (Dick), School of Veterinary Studies, University of Edinburgh, Easter Bush, Midlothian, EH25 9RG UK

## Abstract

The respiratory tract is a key organ for many avian pathogens as well as a major route for vaccination in the poultry industry. To improve immune responses after vaccination of chickens through increased uptake of vaccines and targeting to antigen presenting cells, a better understanding of the avian respiratory immune system is required. Transgenic MacReporter birds were used expressing a reporter gene (*eGFP* or *mApple*) under the control of the *CSF1R* promoter and enhancer in cells of the mononuclear phagocyte (MNP) lineage to visualize the ontogeny of the lymphoid tissue, macrophages and dendritic cells, in the trachea, lung and air sac of birds from embryonic day 18–63 weeks of age. Small aggregates of *CSF1R*-transgene^+^ cells start to form at the openings of the secondary bronchi at 1 week of age, indicative of the early development of the organised bronchus-associated lymphoid tissue. Immunohistochemical staining revealed subpopulations of MNPs in the lung, based on expression of *CSF1R*-transgene, CD11, TIM4, LAMP1, and MHC II. Specialised epithelial cells or M cells covering the bronchus-associated lymphoid tissue expressed *CSF1R*-transgene and type II pneumocytes expressed LAMP1 suggesting that these epithelial cells are phagocytic and transcytose antigen. Highly organised lymphoid tissue was seen in trachea from 4 weeks onwards. Throughout the air sacs at all ages, *CSF1R*-transgene^+^ cells were scattered and at later stages, *CSF1R*-transgene^+^ cells lined capillaries. These results will serve as a base for further functional characterization of macrophages and dendritic cells and their role in respiratory diseases and vaccine responses.

**Electronic supplementary material:**

The online version of this article (10.1186/s13567-018-0598-7) contains supplementary material, which is available to authorized users.

## Introduction

In the poultry industry, vaccines are frequently delivered via spray and aerosol, providing an economical, efficient and reliable method for immunisation of a large number of birds. The respiratory tract is a major portal of entry of pathogens that are of economic or zoonotic importance, such as avian influenza virus (AIV), infectious bronchitis virus (IBV), Newcastle Disease virus and avian pathogenic *Escherichia coli* (APEC). Surprisingly little is known about the ontogeny and function of the immune cells in the avian respiratory tract. Similar to mammals, nonspecific defense mechanisms, such as aerodynamic filtration, mucociliary clearance and antimicrobial substances are the first line of defense of the avian respiratory tract [1–3]. The avian respiratory system differs significantly from mammals. For example, birds have relatively rigid lungs, lack a diaphragm and the lung opens into 9 air sacs that function as bellows, with a unidirectional air flow pattern that affects the deposition of particles [4–6].

**Figure 1:**
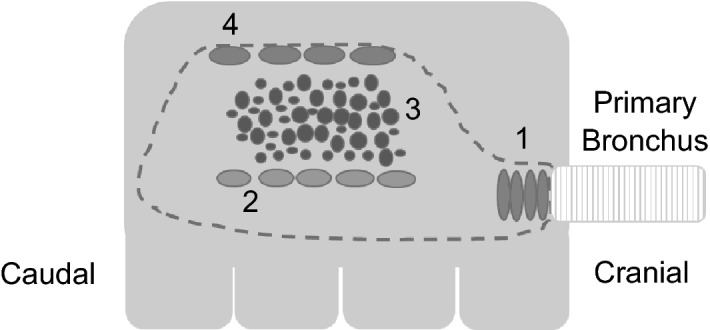
**Schematic overview of the anatomy of the lung.** Frontal plane of the right lung showing the extrapulmonary primary bronchus enters the lung and branches out to secondary bronchi and openings of the medioventral (1), laterodorsal (2), posterior secondary (3), and lateroventral (4) secondary bronchi are depicted (based on Makanya and Djonov [8]). From the secondary bronchi a large number of tertiary bronchi or parabronchi originate.

The lymphoid tissue in the avian lung can be divided into highly organized lymphoid structures such as the bronchus-associated lymphoid tissue (BALT) and diffusely distributed lymphoid cells in the lamina propria around secondary bronchi and in the parabronchi and the interparabronchial connective tissue. In chickens, BALT structures are confined to the openings of the most caudal secondary bronchi (SB) [7], the laterodorsal SB and the posterior SB [8] (Figure 1). Mature BALT structures consist of a distinctive layer of epithelial cells, the lymphoepithelium or follicle-associated epithelium (FAE) [9] overlying primary follicles with densely packed lymphocytes and secondary follicles with germinal centers (GCs) with a subepithelial dome consisting of CD4^+^ T cells, similar to Peyer’s patches and other gut-associated lymphoid tissue (GALT) [10]. CD8^+^ T cells and heterophils are distributed throughout the BALT [1]. BALT appear as early as 2–3 weeks of age and its development is influenced by age and environmental stimuli [7, 11].

To rapidly detect invading pathogens, the respiratory tract is lined with monocytes, macrophages and dendritic cells (DCs), collectively termed mononuclear phagocytes (MNP). Macrophages maintain a low inflammatory environment in the lung, as the infiltration of cells in response to inflammatory stimuli reduces the efficiency of gas exchange, but during infection they immediately induce a response in coordination with epithelial cells and DCs [reviewed in [12, 13]. Polynuclear phagocytes, or granulocytes, and type II pneumocytes (also known as type II alveolar epithelial cells) also display phagocytic capacity, but these cells have distinct functions compared to MNPs. In the mammalian lung during steady state, distinct populations of alveolar macrophages, interstitial macrophages and DCs have been described based on multicolour flowcytometry, but during inflammation this distinction becomes less straight forward [reviewed in 12, 13]. In contrast to mammals, the chicken lung contains very few free-residing macrophages comparable to alveolar macrophages [14]. However, many cells express macrophage and DC markers and are scattered throughout the interstitial tissue of the parabronchial wall and in close contact to the epithelium [15–17]. These cells are expected to play a similar role as mammalian alveolar macrophages and seem to be strategically located at the start of the gas-exchange area to clear the air of inhaled particles before it reaches the thin and vulnerable air capillaries [17].

The trachea in steady state is lined by ciliated pseudostratified columnar epithelium and has a relatively thin lamina propria with large numbers of mucous glands. The ciliated epithelium and mucus create a mucociliary escalator which forms an essential mechanism for entrapment and clearance of particulate material. The lamina propria leukocytes consist mainly of macrophages and an occasional CD4^+^ and CD8^+^ T cells and B cells [18]. After infection with respiratory pathogens, such as IBV, *E. coli* or AIV, the tracheal wall thickens and large infiltrations of macrophages and T cells have been described [19–21].

Little is known about the immune cells that reside within the air sacs, partly owing to their fragility and the low number of cells that can be extracted for ex vivo analysis [19, 22]. The respiratory surface of the air sacs is lined by a simple epithelium, either flat or ciliated, on a basement membrane supported by a thin layer of connective tissue [22, 23] where scattered solitary phagocytes have been reported [24]. The lamina propria of the air sacs contains small capillaries [25], arterioles, venules and lymph vessels [23]. Small lymphoid aggregates have occasionally been seen in the epithelium of the air sacs [11], and they increase in size and number upon infection [22].

The development of transgenic chickens, such as the *CSF1R*-transgenic or MacReporter chicken, enables visualization of the immune system as the reporter gene expression gives a unique macroscopic view of chicken lymphoid structures [26]. *CSF1R*-transgenic chickens express the gene for a fluorescent protein under the control of the *CSF1R* promoter and enhancer, in many hematopoietic cells of the monocyte lineage and at low levels in heterophils [26]. Transgene expression is not a definite marker for all MNP in the chicken as it was recently shown that the Kupffer cells in the liver of MacReporter chickens lack transgene expression, but express *CSF1R* mRNA, indicating an alternative control of CSF1R expression [27]. In this study, we first investigated the ontogeny of the MNPs in the chicken respiratory tract from embryonic day (ED) 18 up to adult birds, and phenotypically characterized cell subpopulations in situ. These transgenic birds enable future studies on the interaction between MNPs and respiratory pathogens, antigen uptake and mucosal targeting of respiratory vaccines.

## Materials and methods

### Chicken lines

Two transgenic chicken lines, collectively named MacReporter chickens, were used in this study. MacRed birds express a modified red fluorescent protein (mApple) and MacGreen birds express an enhanced green fluorescent protein (eGFP) in cells of the mononuclear phagocyte lineage, under the control of the *CSF1R* promoter and enhancer [26]. Birds were bred and reared in floor pens at the National Avian Research Facility, The Roslin Institute, Edinburgh (UK). The animals used for developmental studies and whole mount imaging were vaccinated according to Additional file 1. Animals used for immunohistology were not vaccinated. The chickens were housed in groups and received food and water ad libitum. All birds were considered healthy by physical examination. The age groups studied included MacRed birds at ED18, 1 day, and 1, 2, 4, 8, 10, 16, 30 and 40 weeks of age, and MacGreen birds at 5, 7, 11 and 63 weeks of age (not all ages shown) and were of mixed sexes. Four to six individuals per age group were randomly selected and killed by cervical dislocation, and death was confirmed by decapitation. Animals were housed in premises licensed under a UK Home Office Establishment License (PEL 60/4604) in full compliance with the requirements of the Animals (Scientific Procedures) Act 1986. Breeding of transgenic chickens was carried out under the authority of Project License PPL 70/8940 and application of substances was conducted under PPL 70/7860 with the consent of The Roslin Institute Animal Welfare and Ethical Review Board.

### Tissue preparation

Tissues were collected immediately after death using standard avian necropsy techniques. Both lungs containing the primary bronchi were carefully removed from the coelomic cavity. One of the lungs was incised longitudinally on its ventral aspect, from the extrapulmonary primary bronchus into the intrapulmonary primary bronchus, in order to enable imaging the mucosa of the intrapulmonary primary bronchus and the openings to the SB. The other lung was incised transversally along the second costal sulci. The trachea, from the first tracheal rings to just above the carina, was dissected from the cervical area and two parallel longitudinal incisions were made to expose the tracheal mucosa. The cranial and caudal thoracic air sacs were collected intact and flat using the card-collection technique (Whatman^®^, Grade 54) [28]. After dissection, tissues were kept in a Petri dish on ice, and whole mount fluorescence imaging was immediately performed. Whole mount tissues were evaluated for the presence of fluorescence using the AXIO Zoom.V16 fluorescence stereo zoom microscope (Carl Zeiss Zen pro 2012 software).

### Immunofluorescence

Tissues were fixed in 4% paraformaldehyde for 3–6 h at 4 °C, gently rinsed in phosphate buffered saline (PBS), and overnight immersed in 30% sucrose at 4 °C. Tissue samples were snap frozen in liquid nitrogen and stored at −80 °C until use. Samples were sectioned at 7 µm onto Superfrost Plus slides, air dried in the dark at room temperature (RT) for at least 2–24 h before staining. Sections were blocked for 1 h using 2.5% Skim Milk Powder (SMP, Oxoid), 1% Triton X-100 (Sigma), and 10% normal goat serum or 10% normal horse serum (AMS Biotechnology) in PBS (blocking buffer). All sections were incubated with the primary antibody diluted in blocking buffer at 4 °C overnight and washed in PBS, followed by incubation with the appropriate secondary antibody diluted in blocking buffer and 10 μg/mL of 4′,6-diamidino-2-phenylindole (DAPI; Sigma-Aldrich) for 2 h at RT and washed in PBS. The primary and secondary antibodies used for cell-specific staining are listed in Table 1. Sections were mounted using SlowFade^®^ Gold or using ProLong^®^ Diamond Antifade Mountant (Life Technologies; ThermoFisher Scientific). In each study involving immunofluorescence staining, a tissue section from a chicken that does not express the *CSF1R*-transgene was included as an auto-fluorescence control, and one extra tissue section from the transgenic bird was included and incubated with an unrelated antibody of the same isotype and concentration as the primary antibody as an isotype control for aspecific staining and calibration of the microscopes (see Additional file 2). Images were captured using a Leica DMLB fluorescence microscope (Micro-Manager 1.4.18 software) and confocal images were obtained using an inverted confocal microscope (Zeiss LSM710) and captured using Zeiss Zen (Black) 2012 software.

**Table 1:** **List of primary and secondary antibodies used for immunohistochemistry**

Antibody name	Antigen	Isotype	Supplier	Working concentration
Rabbit anti-GFP Alexa Fluor^®^ 488	eGFP transgene	IgG	Thermo Fischer Scientific	4 µg/mL
Mouse anti-chicken MHCII (clone TAP1)	Class II MHC	IgG2a	DSHB	0.2 µg/mL
Mouse anti-chicken CD11 (clone 8F2)	CD11	IgG1	Kind gift from Dr Sonja Hartle (LMU)	3 µg/mL
Mouse anti-chicken TIM4 (clone JH9)	TIM4	IgG1	In house [27]	2 µg/mL
Mouse anti-chicken LEP100	Chicken lysosomal-associated membrane glycoprotein (LAMP1), present in late endosome and lysosomes	IgG1	DSHB	10 µg/mL
Mouse anti-chicken IgG (clone G-1)	IgY	IgG1	Southern biotech	5 µg/mL
Mouse anti-chicken Bu-1 (clone AV20)	chB6	IgG1	Southern biotech	5 µg/mL
Mouse anti-chicken CD3 (clone CT3)	CD3	IgG1	Southern biotech	5 µg/mL
Jacalin-biotin	*O*-glycosidically linked oligosaccharides	–	Vector Laboratories	10 µg/mL
Phalloidin-Alexa Fluor^®^647	F-actin	–	Thermo Fischer Scientific	0.132 µm
Mouse IgG1 (NC-1390-P)	IgG1 isotype	IgG1	Thermo Fischer Scientific	5 µg/mL
Mouse IgG2a (NC-1391-P)	IgG2a isotype	IgG2a	Thermo Fischer Scientific	5 µg/mL
Donkey anti-rabbit IgG Alexa Fluor^®^ 488	Rabbit IgG	IgG	Invitrogen	2 µg/mL
Goat anti-mouse IgG1 Alexa Fluor^®^ 568	Mouse IgG1	IgG1	Invitrogen	2 µg/mL
Goat anti-mouse IgG2a Alexa Fluor^®^ 568	Mouse IgG2a	IgG2a	Invitrogen	2 µg/mL
Streptavidin- Alexa Fluor^®^ 568	Biotin	–	Biolegend	0.2 µg/mL

DSHB: Developmental Studies Hybridoma Bank; LMU: Ludwig-Maximilian University, Munich, Germany.

### Uptake of antigens

Three-week-old MacGreen birds were administered 0.1 or 1 µm Yellow/Green FluoSpheres™ Carboxylate-Modified Microspheres (Thermofisher) intratracheally using a flexible oral gavage needle (20 × 38 mm, Instech). Alternatively, MacRed animals were administered heat-inactivated APEC engineered to express GFP intratracheally using a flexible oral gavage needle (20 × 38 mm, Instech). The genome sequenced APEC 01 strain (serotype O1:K1:H7 [29]) was transformed with plasmid pFVP25.1 [30] for this purpose. Samples were collected for whole mount and immunofluorescence analysis 30 min, 3 h and 1 day post-administration as previously outlined.

## Results

### Development and distribution of mononuclear phagocytes and germinal centers in the lung of *CSF1R*-transgenic birds

The distribution of *CSF1R*-transgene expressing cells was examined in lungs of MacReporter birds of different ages by whole mount microscopy. These birds were reared in floor pens on wood shavings and routinely vaccinated, albeit via non-respiratory routes (Additional file 1). Therefore, the lymphoid accumulations observed are in part in response to environmental particles and the vaccinations. Although BALT development follows a similar time course in specific pathogen-free and conventionally reared chickens, differences were observed with regard to germinal center (GC) development [7].

**Figure 2:**
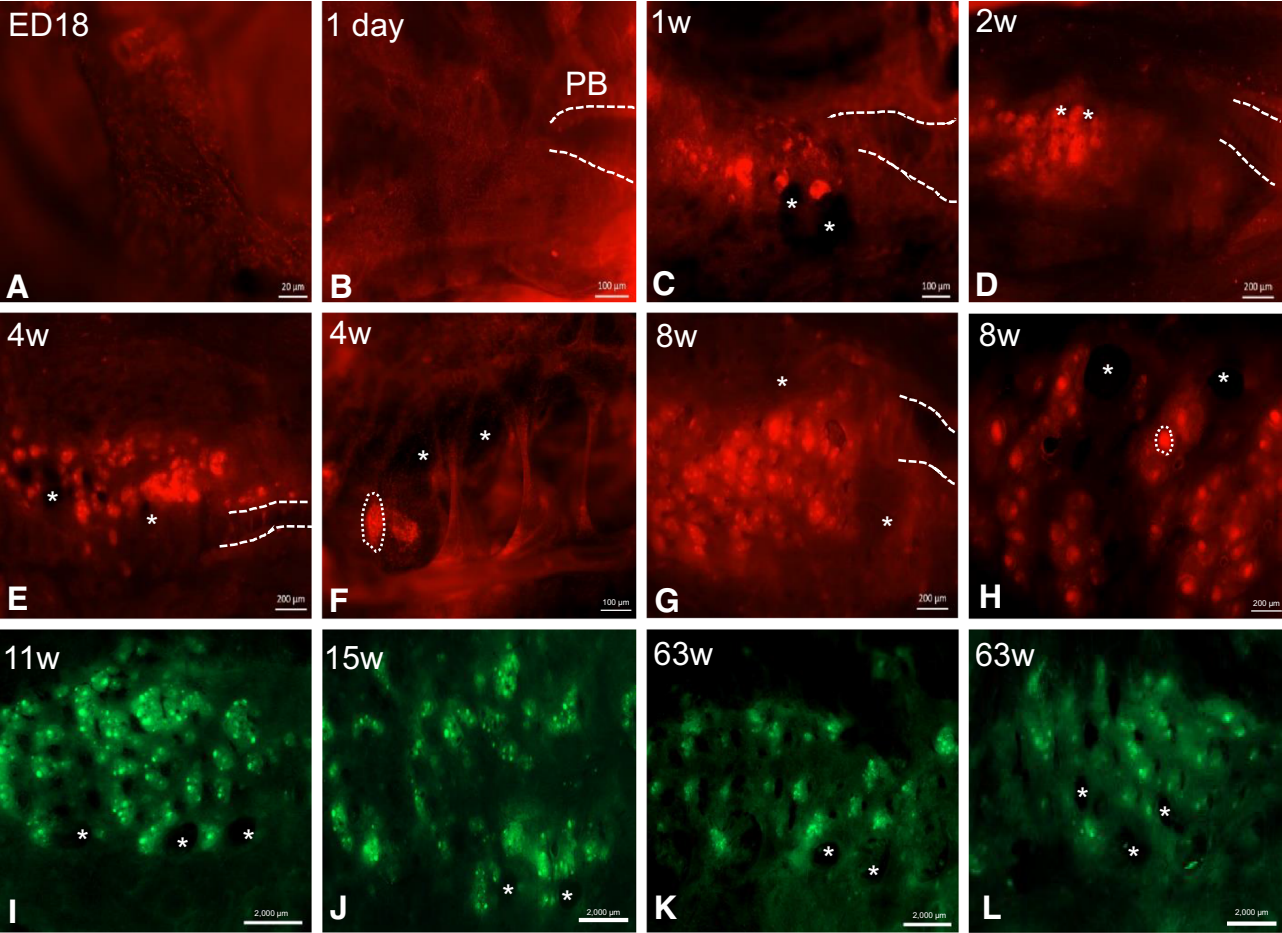
**Development of BALT regions in the intrapulmonary primary and secondary bronchi of** ***CSF1R*-transgenic birds.** Whole mount analysis of the intrapulmonary bronchi of a *CSF1R*-mApple chicken. BALT structures are associated with each opening of the secondary bronchi and increase in size and number with age (**A**–**L**). Intrapulmonary bronchi of older *CSF1R*-eGFP animals show the presence of BALT (**K**–**L**) and the number of lymphoid follicles seemingly reduces in older animals (**K**–**L**). Primary bronchus (PB) is indicated by dashed lines, lymphoid follicles indicated by a dashed circle and openings to the secondary bronchi are marked by an asterisk. w = week/s of age.

In the lung scattered individual *CSF1R*-transgene^+^ cells were distributed over the entire mucosa of the intrapulmonary primary bronchi in all age groups (Figures 2A–L). At ED18 and 1 day of age, there are no signs of organised structures or cellular aggregates of *CSF1R*-transgene^+^ cells (Figures 2A and B). Small aggregations of *CSF1R*-transgene^+^ cells were first observed at 1 week of age (Figure 2C). They are primarily located at the vicinity of openings of laterodorsal and mediodorsal SB. With increasing age these aggregates become larger and more numerous (Figures 2D–J). They are distributed at the vicinity of the laterodorsal, mediodorsal and medioventral openings. From 4 weeks of age onwards, GCs are observed within these lymphoid follicles, and they are characterized by tightly packed aggregates of *CSF1R*-transgene^+^ cells (Figures 2E–H). GCs in older birds are larger with some lymphoid follicles containing several GCs (Figures 2I and J), similar to the Peyer’s patches of the small intestine [1]. Lymphoid follicles residing in the BALT region were occasionally observed in the vicinity of the third and fourth openings of the medioventral SB, and follicles were also present in the vicinity of the openings of the medioventral SB.

In this study, the lateroventral SB were not appreciated, as they do not open directly into the intrapulmonary primary bronchi and are unlikely to be identified using non-casting methods [8]. Moreover, organised lymphoid tissue with and without GCs were observed on the transversal sections of the SB within the parabronchi, indicating that they are not restricted to the openings of the medioventral SB as it enters the parabronchial tissue. The animals used in this study appeared to have BALT structures 1 week post-hatch which were present in birds of up to 63 weeks of age, although the number of follicles seems reduced in older animals this could be attributed to the increase in lung size and follicles being dispersed over a larger surface area (Figures 2K and L).

### Immunofluorescent staining of cell populations in the bronchus-associated lymphoid tissue

**Figure 3:**
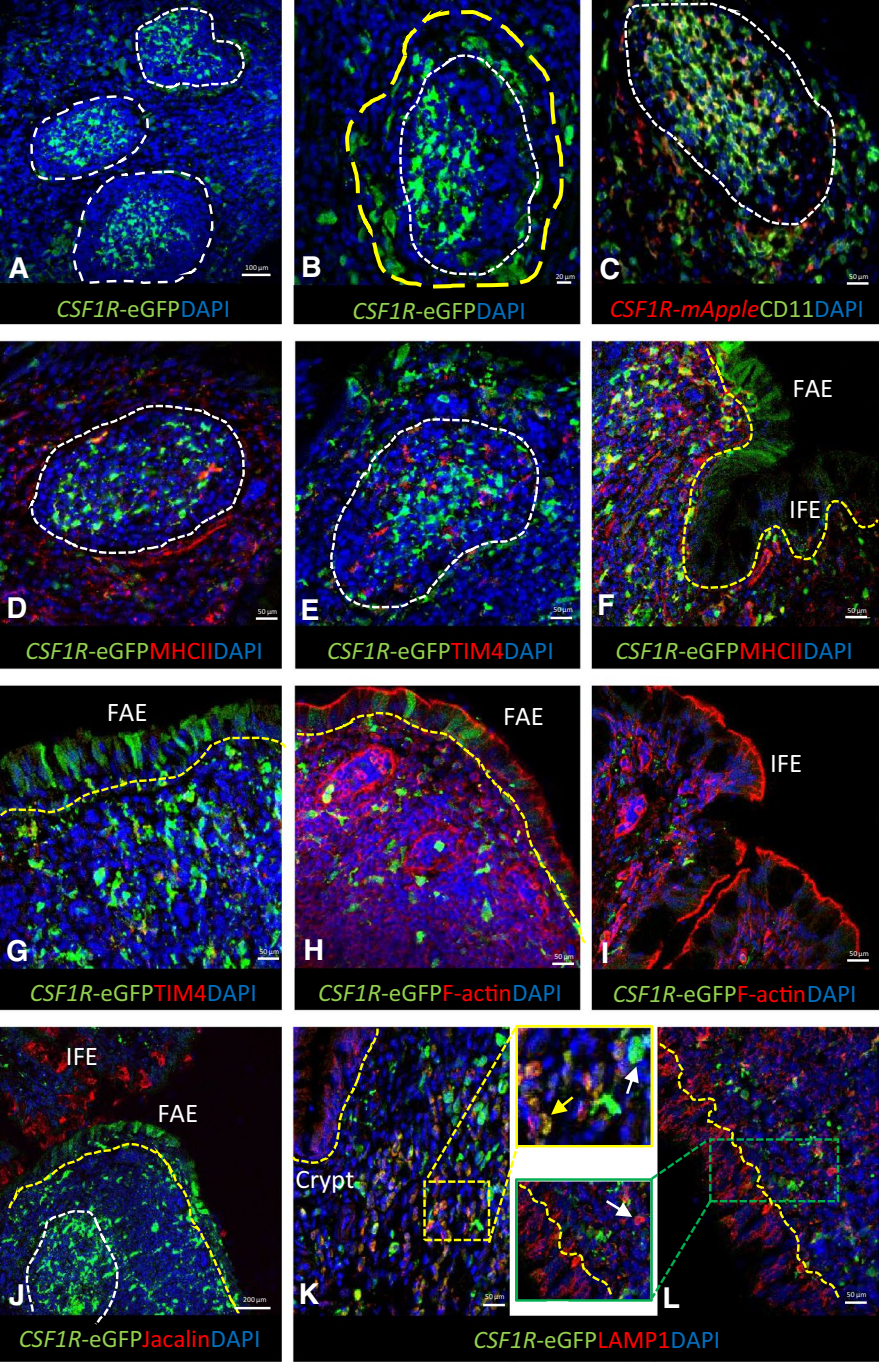
**The BALT region in** ***CSF1R*-transgenic birds.** The BALT of the chicken lung is constitutive, consisting of several GC (**A**) with tightly packed *CSF1R*-eGFP^+^ cells in the light zone and a characteristic halo of MPN surrounding the GC (**B**). In the GC, *CSF1R*-transgene^+^ cells express CD11 (**C**) and lack MHC II (**D**). TIM4^+^ *CSF1R*-transgene^−^ cells are present in the GC known as tingible body macrophages. Outside the GC occasional TIM4^+^ *CSF1R*-transgene^+^ cells were observed (**E**). *CSF1R*-eGFP^+^ cells can be found in regions of the epithelial overlaying the BALT (FAE), called M cells (**F**–**H**). Lung M cells lack MHC II (**F**) and TIM4 (**G**) expression. The M cells express high levels of F-actin (**H**) similar to IFE cells (**I**). M cells lack jacalin binding in contrast to the IFE (**J**). LAMP1 is expressed in *CSF1R*-transgene^+^ (yellow arrow, **K**) and *CSF1R*-transgene^−^ cells (white arrow, **L**). LAMP1 is also highly expressed by epithelial cells (**L**). GC are indicated by white dashed lines with halo of cells identified with a yellow dashed circle, epithelial layer is indicated by yellow dashed lines.

To enable a more in-depth phenotypical analysis of MNP subpopulations, immunostaining of BALT and parabronchi was performed on non-vaccinated animals between 5 and 7 weeks of age. *CSF1R*-transgene^+^ cells were abundantly present in BALT structures and were more tightly packed in the centers or light zone of GCs than in other parts of the lymphoid tissue (Figures 3A and B). This is consistent with previous reports of monocytes/macrophages and DC type cells present in the BALT, which are CVI-ChNL-68.1 and CVI-ChNL-74.2-positive, and express MHC II [1, 31]. chB6^+^ B cells were found to be tightly packed in GCs interspersed with *CSF1R*-transgene^+^ cells, while CD3^+^ T cells were observed to aggregate in poorly demarcated areas within the BALT, in the subepithelial areas and in between GCs (Additional file 2). The remaining areas of the BALT have a more disperse population of both B and T cells (Additional file 2).

GCs have tightly packed *CSF1R*-transgene^+^ cells (Figures 3A and B) known as follicular dendritic cells (FDC) that express CD11, but not MHC II (Figures 3C and D) and have previously shown to express CVI-ChNL-74.3 [31]. FDCs are characterised by trapping immune complexes via Fc receptors (Additional file 2). Their enzyme content is restricted to non-specific esterase and adenosine triphosphate [32, 33]. FDCs are absent in the dark zone or periphery of a GC where the rate of B cell proliferation is high [34]. We observed a large population of CD11^+^ cells to be present throughout the BALT. In GCs, CD11^+^ cells were tightly packed, and virtually all *CSF1R*-transgene^+^ cells were CD11^+^. While most of the *CSF1R*-transgene^+^ cells are CD11^+^, small subpopulations of *CSF1R*-transgene^−^ CD11^+^ and occasional *CSF1R*-transgene^+^ CD11^−^ cells were observed outside the GCs (Figure 3C).

In addition, GCs contain a cell population with remnants of nuclei in their cytoplasm, also known as starry-sky or tingible body macrophages. In the chicken, these cells express TIM4, a receptor for phosphatidylserine, and engulf apoptotic cells. These TIM4^+^ cells lack transgene expression (Figure 3E), but possibly express *CSF1R* transcript as previously described for TIM4^+^ *CSF1R*-transgene^−^ cells in the chicken spleen [27].

The epithelium overlying the BALT consist of the follicle-associated epithelium (FAE) and interfollicular epithelium (IFE). Occasionally, in the FAE, *CSF1R*-transgene^+^ cells were observed that were negative for common APC markers used in this study (Figures 3F and G). These cells likely represent airway M cells whose primary function is to transport mucosal particles to underlying APCs [35, 36] and have recently been described in the avian bursa (Balic & Vervelde, unpublished observations). The FAE and IFE highly express F-actin at the apical side of the cell which likely plays a role in remodeling of the cells during endocytosis (Figures 3H and I) [37]. Differentiation between FAE and bronchial IFE was visualised with the α-D-galactose binding lectin, jacalin. Jacalin does not bind to the FAE, but binds to bronchial IFE cells (Figure 3J), suggesting that the FAE covering the BALT, similar to the epithelium covering the gut-associated lymphoid tissue or Peyer’s patches, differs in detail in relation to their function.

Staining of lysosome-associated membrane glycoprotein-1 (LAMP1/CD107a) was cytoplasmic and granular and correlated with the location of lysosomes, organelles that degrade intracellular material endocytosed by the cell. Scattered LAMP1^+^ cells were found to be distributed within the BALT, and non-GC areas (Figures 3K and L). Almost all LAMP1^+^ cells express the *CSF1R*-transgene, with occasional cells being LAMP1^+^ *CSF1R*-transgene^−^ (Figures 3K and L). LAMP1 was also found to be highly expressed by epithelial cells of the BALT (Figure 3L).

### Immunofluorescent staining of cell populations in the parabronchi

**Figure 4:**
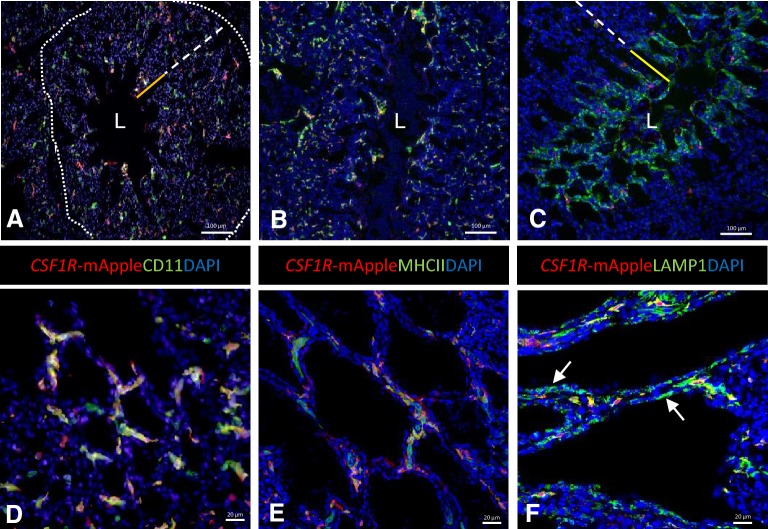
**The parabronchus region in** ***CSF1R*-transgenic birds.** Tertiary bronchi or parabronchi represent the functional unit of gas exchange and consist of a lumen (L), atria (solid yellow line) and infundibulum (dashed white line) surrounded by connective tissue that contains blood vessels and lymphoid follicles (indicated by dotted line). Scattered *CSF1R*-transgene^+^ are located throughout the parabronchus and co-express CD11 (**A**) and MHC II (**B**). LAMP1 is expressed in the majority of the *CSF1R*-transgene^+^ cells and in the pneumocytes in the interatrial septa (**C**). Enlargements of the interatrial septa (**D**–**F**).

Similar to the BALT, non-vaccinated 5–7 week old animals were used to study cells in the parabronchi in more detail. Scattered *CSF1R*-transgene^+^ cells are present throughout the parabronchi, including in the infundibulum and interatrial septa and occasionally observed in the epithelium of parabronchi, around atrial smooth muscle (Figure 4). B and T lymphocytes are scattered throughout the parabronchi with T cells found to be in close proximity with *CSF1R*-transgene^+^ cells (Additional file 2).

More in depth analysis of the MNP subpopulations shows scattered CD11^+^ cells present throughout the parabronchi, including interatrial septa (Figures 4A and D). All *CSF1R*-transgene^+^ cell expressed CD11 and a subpopulation of CD11^+^ cells were observed to be *CSF1R*-transgene^−^. Clusters of CD11^+^ *CSF1R*-transgene^−^ cells were also observed in association with blood vessels in the connective tissue that separates two adjoining parabronchial lobules. Individual MHC II^+^ cells were found to be distributed throughout the parabronchi, including in the atrial septa (Figures 4B and E). Most of the MHC II^+^ cells are also *CSF1R*-transgene^+^, with some scattered MHC II^+^ *CSF1R*-transgene^−^ cells in the lamina propria, likely being B cells.

Furthermore, we observed perivascular lymphoid aggregates of CD11^+^ cells in the parabronchi which have previously been reported [11]. Occasionally, MHC II^+^ cells formed small aggregates and are likely DCs and B cells. The antibodies used in this study do not suffice to distinguish between macrophages and DCs because both cell populations can express the *CSF1R*-transgene, MHC II, CD11, and LAMP1.

A majority of the *CSF1R*-transgene^+^ cells expressed LAMP1 and these cells were located in the atrial septa and infundibulum. Pneumocytes in the interatrial septa were *CSF1R*-transgene^−^ but strongly positive for LAMP1, in contrast, epithelium lining the infundibula and air capillaries were negative for LAMP1 (Figures 4C and F).

#### Development and distribution of MNP and GCs in the trachea of CSF1R-transgenic chickens

**Figure 5:**
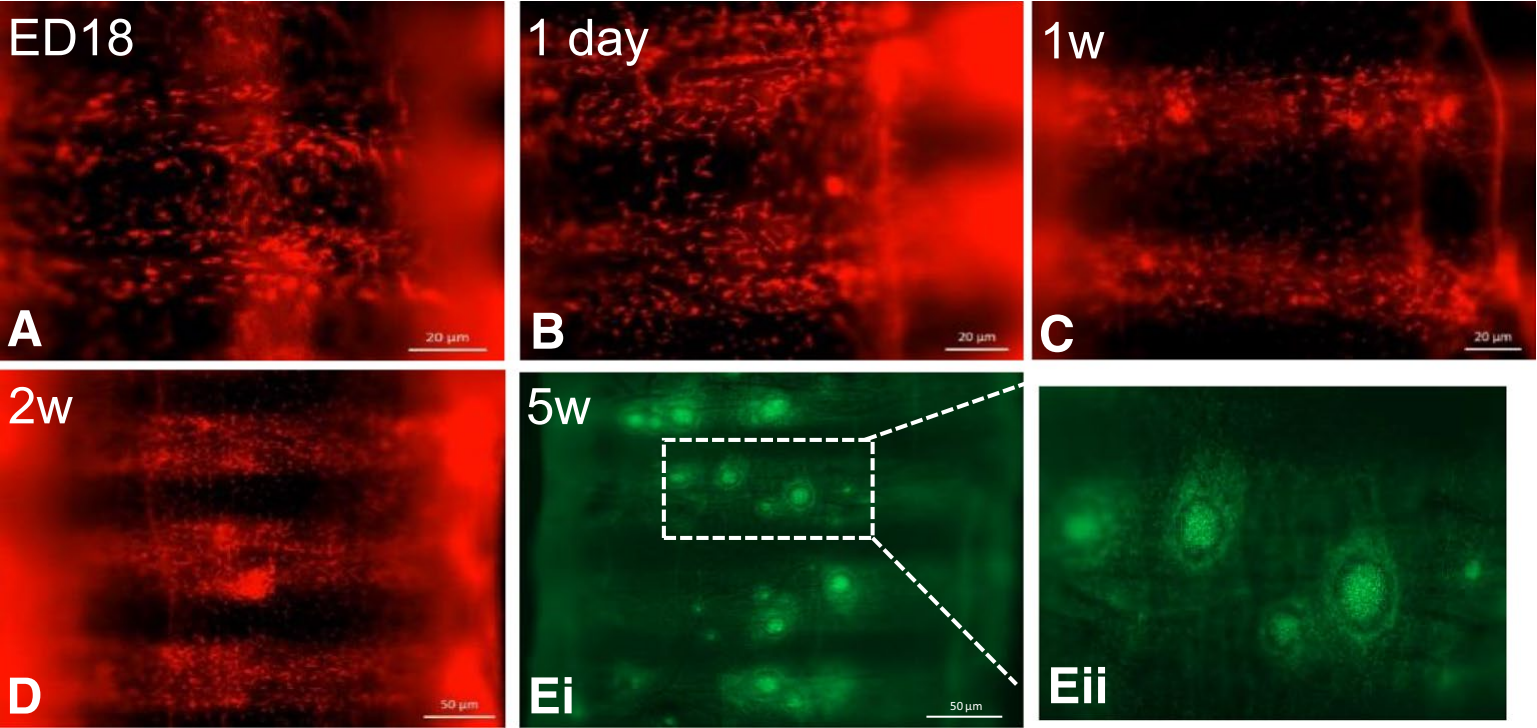
**Development and distribution of MNP and GCs in the trachea of** ***CSF1R*-transgenic birds.** *CSF1R*-mApple^+^ cells are distributed throughout the tracheal mucosa from ED18, forming small aggregates from 1 week of age (**A**–**D**). At 5 weeks post-hatch, mature GC with tightly packed *CSF1R*-eGFP^+^ cells are surrounded by a halo of *CSF1R*-transgene^+^ cells (**Ei**–**Eii**). w = week/s of age.

The distribution of *CSF1R*-transgene expressing cells was examined in trachea of MacReporter chickens of different ages. Scattered individual *CSF1R*-transgene^+^ cells were observed to be distributed throughout the tracheal mucosa, in all age groups (Figures 5A–E). At ED18 the formation of tracheal epithelium is evident and becomes more defined 1 day post-hatch (Figures 5A and B). Although rarely observed at 1 day of age, small aggregates of *CSF1R*-transgene^+^ cells were observed throughout the trachea at 1-week of age (Figure 5C). These lymphoid aggregates increase in size and number as the bird increases in age (Figures 5D and E). From 4 weeks onwards, areas with multiple lymphoid follicles were observed on the tracheal mucosa, containing tightly packed *CSF1R*-transgene^+^ cells and a clear halo, which become larger and more numerous as the bird ages (Figure 5E). Scattered individual *CSF1R*-transgene^+^ cells were abundantly present in tracheal lamina propria (Figure 5).

#### Development and distribution of MNP and GCs in air sacs of CSF1R-transgenic chickens

**Figure 6:**
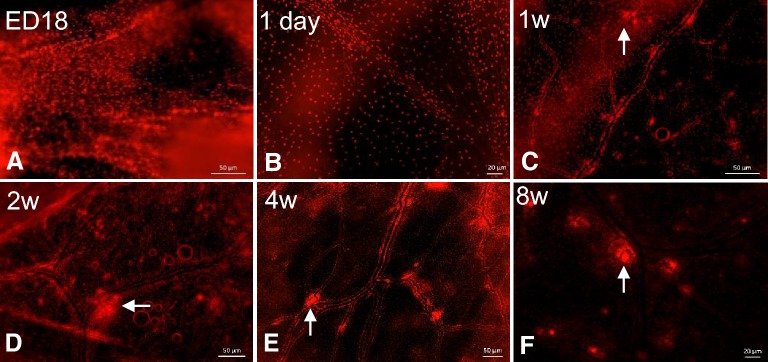
**The development of MNP and GC in air sacs of** ***CSF1R*-transgenic birds.** Whole mount images show that *CSF1R*-mApple^+^ cells are distributed throughout the air sac on ED18 (**A**) and begin to form a pattern 1 day post-hatch (**B**). By 1–2 weeks of age the air sacs have developed an organised distribution that consist of MNP lining the vessels and early lymphoid aggregate formations at the bifurcations of the capillaries (arrows **C**, **D**). By 4 weeks of age these lymphoid aggregates have developed into characteristic GC (arrows **E**, **F**). w = week/s of age.

The distribution of *CSF1R*-transgene expressing cells was examined in the air sac of MacReporter chickens of different ages (Figure 6). Scattered individual *CSF1R*-transgene^+^ cells were distributed throughout the air sacs in all age groups (Figures 6A–F). No cellular aggregation or organized structures were observed at ED18 or 1 day of age, but small aggregations of *CSF1R*-transgene^+^ cells were observed in 1-week-old chicks. Interestingly, at 1 day post-hatch *CSF1R*-transgene^+^ cells begin to form a pattern and clustering can be observed in the vicinity of capillary formation (Figure 6B). The lymphoid aggregates are primarily located at the vicinity and bifurcation of capillaries (Figure 6C). These *CSF1R*-transgene^+^ cellular aggregates increase in size and number with age and are frequently observed at 2 and 4 weeks of age (Figures 6D and E). At 8 weeks of age, many of these aggregates have GCs, characterized by tightly packed aggregates of *CSF1R*-transgene^+^ cells surrounded by a clear halo (Figure 6F).

**Figure 7:**
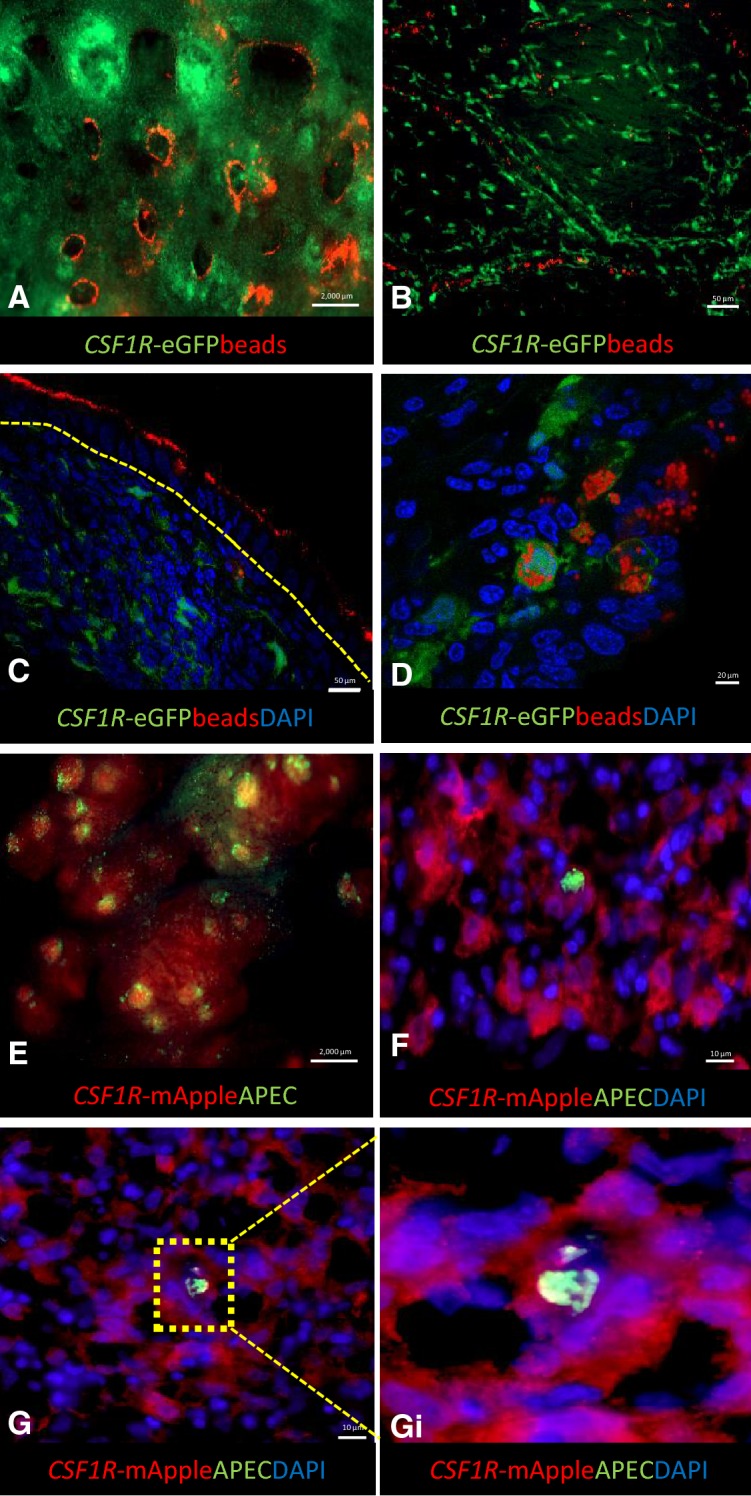
**Applications of the** ***CSF1R*-transgenic birds to study the avian respiratory-associated immune responses.** Intratracheal application of red carboxlylated fluorescence beads in a MacGreen reporter bird (**A**–**D**) and APEC-*GFP* in a MacRed reporter bird (**E**–**F**). Thirty mins post-intratracheal administration, beads (1 µm) are deposited at the openings of the SB (**A**). At 3 h post administration, the beads are co-located with the capillaries of air sacs (**B**). Visualisation of binding and phagocytosis of particles in the lung demonstrated by binding of beads to the epithelial cells of the BALT (**C**; 0.1 µm beads, thirty min post-administration,) and intracellular beads in *CSF1R*-eGFP^+^ cells (**D**; 1 µm beads, 3 h post-administration). An example of host–pathogen interactions is demonstrated in **E**–**F**. Thirty min after intra-tracheal administration, bacteria can be found bound to FAE of the lung (**E**) and APEC-*GFP* uptake by *CSF1R*-mApple^+^ cells in the parabronchus can be visualized 1 day post-infection (**F**–**G**–**Gi**). The epithelial layer is indicated by yellow dashed line.

To demonstrate the potential utility of MacReporter chickens in dissecting host–pathogen interactions, antigen-uptake and vaccine targeting, pilot studies were undertaken using fluorescently labelled beads and heat-inactivated APEC-GFP. Figure 7 shows the deposition of intratracheally administered red fluorescent beads in the openings to the SB, in the air sac, and uptake of fluorescent beads by respiratory epithelium and by MNPs (Figures 7A–D). We were also able to demonstrate colocalisation of APEC-GFP and lymphoid follicle in the BALT area and uptake in the parabronchus of APEC-GFP in *CSF1R*-transgene^+^ cells (Figures 7F and G). Ongoing studies are examining the cell tropism of APEC strains representing dominant serogroups and cellular responses by RNA sequencing of sorted infected cells.

## Discussion

The advantage of using MacReporter chickens is that the development of the lymphoid structures can be assessed ex vivo using whole mount imaging of the entire tissue without the need for tissue preparation and histology. Moreover, the entire trachea and lung can be imaged, lowering the risk of missing smaller focal structures and enabling to dissect specific areas of interest. By analyzing a number of developmental stages during chicken growth, we observed the presence and expansion of lymphoid aggregates consisting of *CSF1R*-transgene^+^ cells in the respiratory tract from vaccinated and non-vaccinated animals.

For the first time the lymphoid aggregates in the whole avian lung were visualised via whole mount microscopy. This application allowed for the visualisation of numerous *CSF1R*-transgene^+^ cellular aggregates in the vicinity of openings to the laterodorsal and mediodorsal SB. The presence of constitutive BALT has been described in chickens, rats and rabbits [7]. In humans, BALT can be found in childhood but regresses into adulthood. However, its constitutive presence is still controversial [38, 39]. A related BALT-like structure known as inducible BALT (iBALT), an ectopic lymphoid tissue, can be found upon inflammation or infection in both mice and humans and appears throughout the lung [39, 40]. In our study vaccinated and non-vaccinated animals reared under conventional conditions presented no differences in the presences of BALT structures albeit vaccinated animals may have more active immune system compared to unvaccinated animals. In chickens, ectopic lymphoid tissues appear in the lung after infection with, for example, avian influenza virus (Vervelde and Reemers, unpublished observations). The development of the BALT in the chicken lung was progressive and consistent with those previously reported [11], with lymphoid follicles and the number of follicles associated with each opening of SB increasing in size and number with age. In contrast to previous studies, in the MacReporter birds we saw small aggregates were already present in 1-week-old chicks. The earlier detection might have been due the fact that animals were vaccinated via the drinking water 1 day post-hatch, but more likely because we could analyse the complete lung instead of histological sections only. Mature BALT structures with B and T cell areas and GCs were observed from 4 weeks of age onwards. Interestingly, lymphoid follicles were also present around the medioventral SB in some of the birds. These are the most cranial SB, and are composed of four large openings in the genus *Gallus* [8]. This is in contrast with previous literature [7], which only reports BALT in the vicinity of the most caudal SB (the laterodorsal and posterior SB).

We examined the MNPs and epithelial cells of the BALT and parabronchi of the lung from 5 to 7 week old non-vaccinated birds reared under conventional conditions. Using immunofluorescence staining, the *CSF1R*-transgene^+^ lymphoid aggregates observed via whole mount microscopy, were also present in non-vaccinated animals and were tightly packed GCs consisting of B cells, FDC and tingible body macrophages, distinguished based on their Bu-1 (chB6) and TIM4 staining and *CSF1R*-transgene^+^ expression. The presence of multiple GCs in the BALT region of non-vaccinated animals is similar to earlier findings that SPF and conventionally reared birds had no difference in BALT development [7]. LAMP1 staining colocalised with *CSF1R*-transgene expression but was also found in cells lacking *CSF1R*-transgene expression. These cells may represent phagocytic cells that express LAMP1 in their cytotoxic granules that do not express the transgene, such as natural killer cells [41] or express the transgene at low levels, such as granulocytes [26]. Respiratory MNPs in the parabronchi have been described previously with CD11^+^, KUL01^+^ and DEC205^+^ cells located in the interstitial tissue of the primary bronchus wall and CD11^+^ and KUL01^+^ cells located in interatrial septa [17]. Although the present study did not distinguish between macrophages and DCs, the MacReporter chicken allow for the appreciation of the location and quantity of MNP in the BALT and parabronchi of unvaccinated healthy animals. We also observed the presence of M cells in the FAE overlaying the BALT. Previous reports have identified these cells by transmission electron microcopy in the turkey [11] and failed to identify them in chicken lung by SEM [7]. In our studies these cells were found to express the *CSF1R*-transgene and lack jacalin binding in contrast to the IFE overlaying the BALT structure. This observations makes BALT-associated M cell identification and study more accessible. M cells can mediate infection by bacteria [35, 42] and viruses [43] and may represent a route to infection in chickens as observed in mammals [35, 36].

In the lung parabronchi, pneumocytes in the interatrial septa were found to be positive for LAMP1 staining but lacked *CSF1R*-transgene expression. The LAMP1 staining in epithelial cells lining the atria suggests that these cells are able to phagocytose and process antigens. These lysosomal bodies in avian pneumocytes have been previously described [44] and the cells were classified as type II pneumocytes [45]. Besides being LAMP1^+^, they also express the FDC marker CVI-ChNL-74.3 [46]. The limited number of highly mobile free residing macrophages in the lumen maybe correlated to the fact that the avian epithelium immediate to the respiratory surface is strongly phagocytic [45]. In addition to the airway, M cells in the epithelium of the primary bronchus expressing *CSF1R*-transgene, these type II pneumocytes also express molecules in common with MNPs. Similarly, mouse, sheep, and human type II pneumocytes and MNPs express DC-LAMP/CD208 [47] and RAGE [48]. Small aggregates of MHC II^+^ cells are occasionally observed in the parabronchi indicating that, along with the BALT, the parabronchi play an important role in the immune response of the avian lung. Scattered CD4^+^ and CD8α^+^ cells are also present in the parabronchi and occasionally forming subepithelial aggregates of variable sizes. Perivascular clusters of B and T cells are observed, and in older birds occasionally have GCs (unpublished observations) [22]. Together with macrophages, T cell numbers increase rapidly after infection or vaccination with respiratory viruses or bacterial infections [19, 21, 43, 49]. The combination of free residing macrophages, phagocytic epithelium, the numerous macrophages and DC in the underlying tissue and the fact that these cells can been mobilised rapidly after infection ensures an effective cellular defense system in the chicken lung.

The development of GCs in the trachea was observed using the MacReporter animals. From ED18 up to 8 weeks post-hatch, the accumulation of tightly packed areas with multiple follicles increased with age. Changes in MNP in the trachea have been reported after infection with e.g. IBV, *E. coli* [19, 49] and ITLV [50]. A similar pattern of lymphoid accumulation during development was also evident in the avian air sacs. Using electron microscopy, Bezuidenhout reported similar findings to this report where perivascular aggregation of leucocytes (macrophages, heterophils, lymphocytes, plasma cells, monocytes and mast cells) were observed in the air sacs [23]. The perivascular aggregation of *CSF1R*-transgene^+^ cells in the air sac could indicate that these cells play an important role in the induction of the local immune response as previously seen after infection with IBV and *E. coli* [19, 22]. Our pilot studies using inert beads and inactivated APEC bacteria show the potential of the MacReporter birds to investigate the respiratory immune system during infection and provide the tools to better investigate host–pathogen interactions and vaccine targeting.

In summary, we demonstrated the potential of *CSF1R*-transgenic MacReporter birds to visualize lymphoid tissue in the respiratory tract of birds of all ages using whole mount imaging and immunofluorescent staining. In all tissues, trachea, lung and air sac, the development was progressive, with the number and size of lymphoid follicles increasing with age. It should be noted that some animals were vaccinated via drinking water which may play a role in the early detection and abundance of follicles observed in this study. The expression of the transgene enabled us to visualize organized lymphoid tissue at an earlier age and especially at different locations than previously published. The results presented in this study will serve as a base for further functional characterization of MNP, macrophages and DC subpopulations in uptake processing and presentation of antigens to T and B cells and the role of MNP in respiratory diseases such as *E. coli*, IBV and avian influenza.

## Additional files

**Additional file 1.** **Vaccination regime at the National Avian Research Facility, Edinburgh, UK.** Routine vaccination regime of birds used for the Visualisation and characterisation of mononuclear phagocytes in the chicken respiratory tract using *CSF1R*-transgenic chickens.

**Additional file 2.** **Location of B cells, T cells and follicular dendritic cells (FDC) in the lung of MacReporter chickens.** The BALT region of 5 to 7 week old non-vaccination animals were analysed for B, T and FCD cells. Isotype controls were used to standardise the microscope and examine aspecific binding before acquiring images (A-B). The GC of MacReporter animals are tightly packed with Bu1^-^*CSF1R-*eGFP^+^ FDC cells and Bu1^+^*CSF1R*-eGFP^-^ B cells (C) with few Bu1^+^ B cells found in the parabronchi (F). CD3^+^ T cells are disperse within and outside the GC (D) and parabronchi (G). *CSF1R*-eGFP^+^ FDC cells express Fc receptors and trap immunoglobulin by expressing IgY (E) and CSF1R-eGFP^+^ IgY^+^ FDC are rarely detected out with the GC, BALT region of the lung. GC are indicated by white dashed lines.

## Abbreviations

AIV: avian influenza virus
APC: antigen presenting cells
APEC: avian pathogenic *Escherichia coli*
BALT: bronchus associated lymphoid tissue
CSF1R: colony stimulating factor 1 receptor
DC: dendritic cell
ED: embryonic day of incubation
eGFP: enhanced green fluorescent protein
FAE: follicle associated epithelium
FDC: follicular dendritic cells
GALT: gut-associated lymphoid tissue
GC: germinal center
IBV: infectious bronchitis virus
IFE: interfollicular epithelium
LAMP1: lysosomal-associated membrane protein 1
MNP: mononuclear phagocytes
PB: primary bronchus
PBS: phosphate buffered saline
SB: secondary bronchus
