# Supplementary material for: Visualisation and characterisation of mononuclear phagocytes in the chicken respiratory tract using CSF1R-transgenic chickens
Source: Vet Res. 2018 Oct 10;49:104. doi: 10.1186/s13567-018-0598-7 (PMC6389226; doi:10.1186/s13567-018-0598-7)
Supplement: Supplementary file 1 — Additional file 1. Vaccination regime at the National Avian Research Facility, Edinburgh, UK. Routine vaccination regime of birds used for the Visualisation and characterisation of mononuclear phagocytes in the chicken respiratory tract using CSF1R-transgenic chickens. [file 13567_2018_598_MOESM1_ESM.doc]

| **Disease** | **Vaccine** | **Age** | **Route** |
| --- | --- | --- | --- |
| Marek’s Disease | Marek’s Disease Vaccine (Rispens CVI 988) | 1 day | Intramuscular |
| Coccidiosis | Hipracox | 1-7 days | Drinking water |
| Newcastle Disease | Poulvac NDW (Clone 30) | 1-3 weeks | Drinking water |
| Infectious Bronchitis | Poulvac IB Primer (H120 and D274) |  |  |
| Infectious Bursal Disease | Bursine (D78/IBD) | 4-6 weeks | Drinking water |
| Chicken Anemia Virus | Nobilis CAV P4 | 6-9 weeks | Drinking water |
| Infectious Laringotracheitis | Poulvac ILT  (Salsbury strain 146) | 6-12 weeks | Eye drops |
| Infectious Bronchitis | Nobilis IB 4/91 |  | Drinking water |
| Newcastle Disease | Poulvac NDW(Clone 30) |  |  |
| Infectious Bursal Disease | Bursine (D78/IBD) |  |  |
| Avian Encephalomielytis | Encefal-vac (Calnek 1143) | 13-14 weeks | Drinking water |
| Avian Pneumovirus | [Nobilis RT + IBmulti + ND +](http://www.merck-animal-health.com/species/poultry/live-vaccines.aspx) EDS | 16+ weeks | Intramuscular |
| Infectious Bronchitis |  |  |  |
| Newcastle Disease |  |  |  |
| Egg Drop Syndrome |  |  |  |
