# Supplementary figures and images for: Visualisation and characterisation of mononuclear phagocytes in the chicken respiratory tract using CSF1R-transgenic chickens
Source: Vet Res. 2018 Oct 10;49:104. doi: 10.1186/s13567-018-0598-7 (PMC6389226; doi:10.1186/s13567-018-0598-7)

Isotype Controls

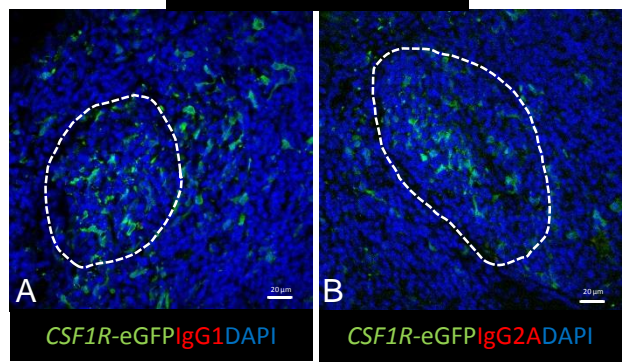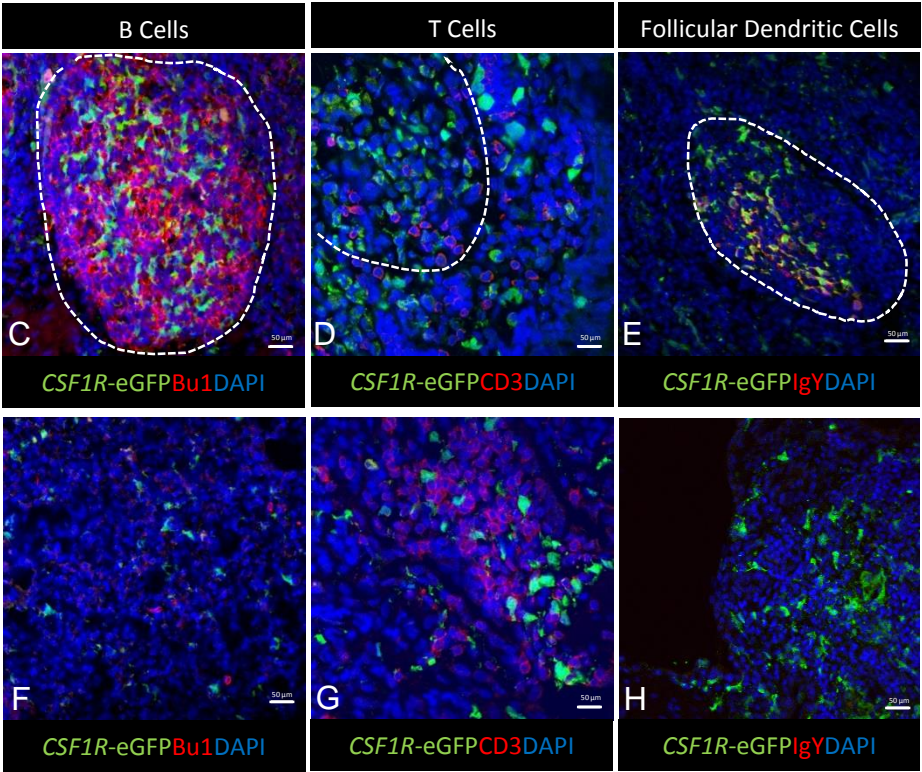

Supplement: Supplementary file 2 — Additional file 2. Location of B cells, T cells and follicular dendritic cells (FDC) in the lung of MacReporter chickens. The BALT region of 5 to 7 week old non-vaccination animals were analysed for B, T and FCD cells. Isotype controls were used to standardise the microscope and examine aspecific binding before acquiring images (A-B). The GC of MacReporter animals are tightly packed with Bu1-CSF1R-eGFP+ FDC cells and Bu1+CSF1R-eGFP- B cells (C) with few Bu1+ B cells found in the parabronchi (F). CD3+ T cells are disperse within and outside the GC (D) and parabronchi (G). CSF1R-eGFP+ FDC cells express Fc receptors and trap immunoglobulin by expressing IgY (E) and CSF1R-eGFP+ IgY+ FDC are rarely detected out with the GC, BALT region of the lung. GC are indicated by white dashed lines. [file 13567_2018_598_MOESM2_ESM.pdf]
